# Causality between sarcopenia and diabetic nephropathy: a bidirectional Mendelian randomization study

**DOI:** 10.3389/fendo.2023.1188972

**Published:** 2023-05-22

**Authors:** Linan Ren, Yao Wang, Feng Ju, Meixin Sun, Xiaokun Gang, Guixia Wang

**Affiliations:** ^1^ Department of Endocrinology and Metabolism, First Hospital of Jilin University, Changchun, Jilin, China; ^2^ Department of Orthopedics, The Second Hospital of Jilin University, Changchun, Jilin, China; ^3^ Department of Orthopedics, Yuci District People’s Hospital, Yuci, Shanxi, China

**Keywords:** appendicular lean mass, grip strength, walking speed, diabetic nephropathy, causality

## Abstract

**Background and purpose:**

Observational studies have shown that sarcopenia and diabetic nephropathy (DN), are closely related; however, the causal relationship is unclear. This study aims to address this issue using a bidirectional Mendelian randomization (MR) study.

**Methodology:**

We data from genome-wide association studies including appendicular lean mass (n = 244,730), grip strength (right: n = 461,089, left: n = 461026), walking speed (n = 459,915), and DN (3283 cases and 181,704 controls) to conduct a bidirectional MR study. First, we conducted a Forward MR analysis to evaluate the causality of sarcopenia on the risk of DN from the genetic perspective with appendicular lean mass, grip strength, and walking speed as exposure and DN as the outcome. Then, DN as the exposure, we performed a Reverse MR analysis to determine whether DN impacted the appendicular lean mass, grip strength, and walking speed of the appendices. Finally, a series of sensitivity studies, such as heterogeneity tests, pleiotropy evaluations, and Leave-one-out analyses, were conducted to assess the MR analysis’s accuracy further.

**Results:**

According to a forward MR analysis, a genetically predicted decrease in appendicular lean mass is associated with an increased risk of developing DN risk (inverse variance weighting[IVW]: odd ratio [OR] = 0.863, 95% confidence interval [CI] 0.767-0.971; P = 0.014). According to reverse MR results, grip strength decreased as DN progressed (IVW: right β = 0.003, 95% CI: - 0.021 to - 0.009, P = 5.116e-06; left β = 0.003, 95% CI: - 0.024 to - 0.012, P = 7.035e-09). However, the results of the other MR analyses were not statistically different.

**Conclusion:**

Notably, our findings suggest that the causal relationship between sarcopenia and DN cannot be generalized. According to analysis of the individual characteristic factors of sarcopenia, reducing in appendicular lean mass increases the risk of developing DN and DN is linked to reduced grip strength. But overall, there is no causal relationship between sarcopenia and DN, because the diagnosis of sarcopenia cannot be determined by one of these factors alone.

## Introduction

Loss of muscle mass and function is a hallmark of the skeletal muscle disease sarcopenia. It might make older adults more likely to experience adverse outcomes such as falls, functional decline, weakness, and death (1). The consensus is that measuring or diagnosing sarcopenia may be possible by grip strength, appendicular lean mass, walking speed. The European Working Group on Sarcopenia in the Elderly, the Asian Working Group on Sarcopenia 2019, and the US Sarcopenia Definition and Outcomes Consortium have developed a series of diagnostic and treatment consensus for sarcopenia (2–4).

Sarcopenia has been described in various pathological conditions, and in the elderly population, according to several studies (5–7). Recently, there has been a lot of attention regarding the relationship between sarcopenia and DN. Sarcopenia and DN may have a common pathological basis in insulin resistance and endothelial dysfunction. Insulin resistance causes protein degradation, inhibits protein biosynthesis and induces muscle growth inhibitors, all contributing to the loss of skeletal muscle. Loss of skeletal muscle also exacerbates insulin resistance (8–10). Similarly, insulin signaling is crucial for in podocyte viability and renal tubular. Insulin’s effects on the vascular system and kidneys are also impaired when insulin resistance occurs, in conditions like obesity and type 2 diabetes (T2DM) (11, 12). According to a meta-analysis’s results, endothelial dysfunction is one possible underlying mechanism of sarcopenia (13). Likewise, Leung et al. suggested that renal microangiopathy begins with endothelial cell injury (14). According to several observational studies, sarcopenia is strongly associated with DN (15–17). Huang et al. showed that sarcopenia is an independent risk factor for the deterioration of DN, which promotes the development and progression of DN (15). However, a study showed that DN might be a possible risk factor for sarcopenia (16). This study used Mendelian randomization (MR) to address this debate. Additionally, this study will offer a theoretical basis for preventing two diseases because sarcopenia and DN are common comorbidities in elderly patients with diabetes.

The advantage of MR is that it overcomes the confounding bias and reverse causality inherent in observational studies (18). In addition, it uses a genetic variation to measure the causality of disease-related risk factors. The current study established a causal relationship between sarcopenia and DN by obtaining readily accessible human genetic data for MR analysis. Appendicular lean mass, grip strength, and walking speed, were the three sarcopenia parameters used to generate MR estimates. In this study, we first examined the clinical phenotype of sarcopenia to investigate its effects on DN. Furthermore, we examined the inverse causality between sarcopenia and DN used the prevalence of DN as an exposure.

## Materials and methods

### MR design

**Figure 1:**
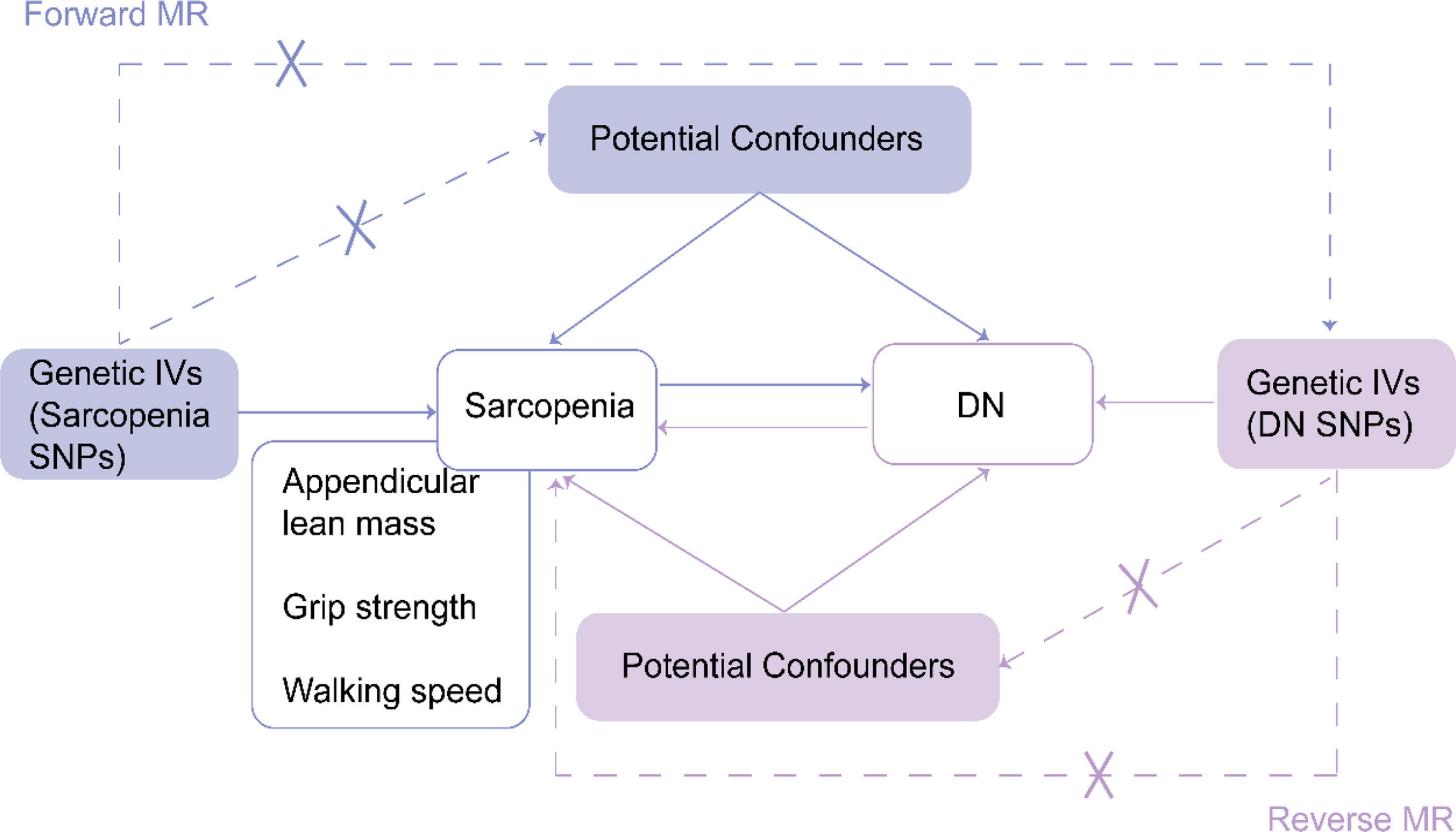
Design of MR analysis of the causal link between sarcopenia and DN. IVs, instrumental variants; SNP, single nucleotide polymorphism; DN: diabetic nephropathy. The flow chart is derived from Liu et al. (19).

MR analysis was performed using instrumental variables (IVs) based on genetic variation. The correlation assumption, independence assumption, and exclusionary restriction assumption are the three essential assumptions of MR studies. Liu et al. have provided the specifics of each in detail (19). **Figure 1** depicts the MR design for this study.

### Data sources description

Genome-wide association studies (GWAS) data, which included 244,730 European samples and 18,164,071 single nucleotide polymorphisms (SNPs) (20). A total of 461,089, 461,026, and 459,915 European samples’ worth of IVs related to grip strength (including right-hand grip strength and left-hand grip strength) and walking speed were obtained from UKBiobank. Additionally, you can get the GWAS summary statistics for DN from the IEU Open GWAS project download, which includes 3283 European cases and 181,704 European controls. **Table 1** displays comprehensive explanation the data involved in the study.

**Table 1:** A detailed description of the GWAS data involved in this study.

Phenotype	Races	Sizes of sample	Years	No. of SNPs	Website
DN	European	3283 cases and 181,704 controls	2021	16,380,336	https://gwas.mrcieu.ac.uk/datasets/finn-b-DM_NEPHROPATHY_EXMORE/
Appendicular lean mass	European	244,730	2020	18,164,071	https://gwas.mrcieu.ac.uk/datasets/ebi-a-GCST90000027/
Grip strength (Right)	European	461,089	2018	9,851,867	https://gwas.mrcieu.ac.uk/datasets/ukb-b-10215/
Grip strength (Left)	European	461,026	2018	9,851,867	https://gwas.mrcieu.ac.uk/datasets/ukb-b-7478/
Walking speed	European	459,915	2018	9,851,867	https://gwas.mrcieu.ac.uk/datasets/ukb-b-4711/

### Selection of instrumental genetic variables for sarcopenia

**Figure 2:**
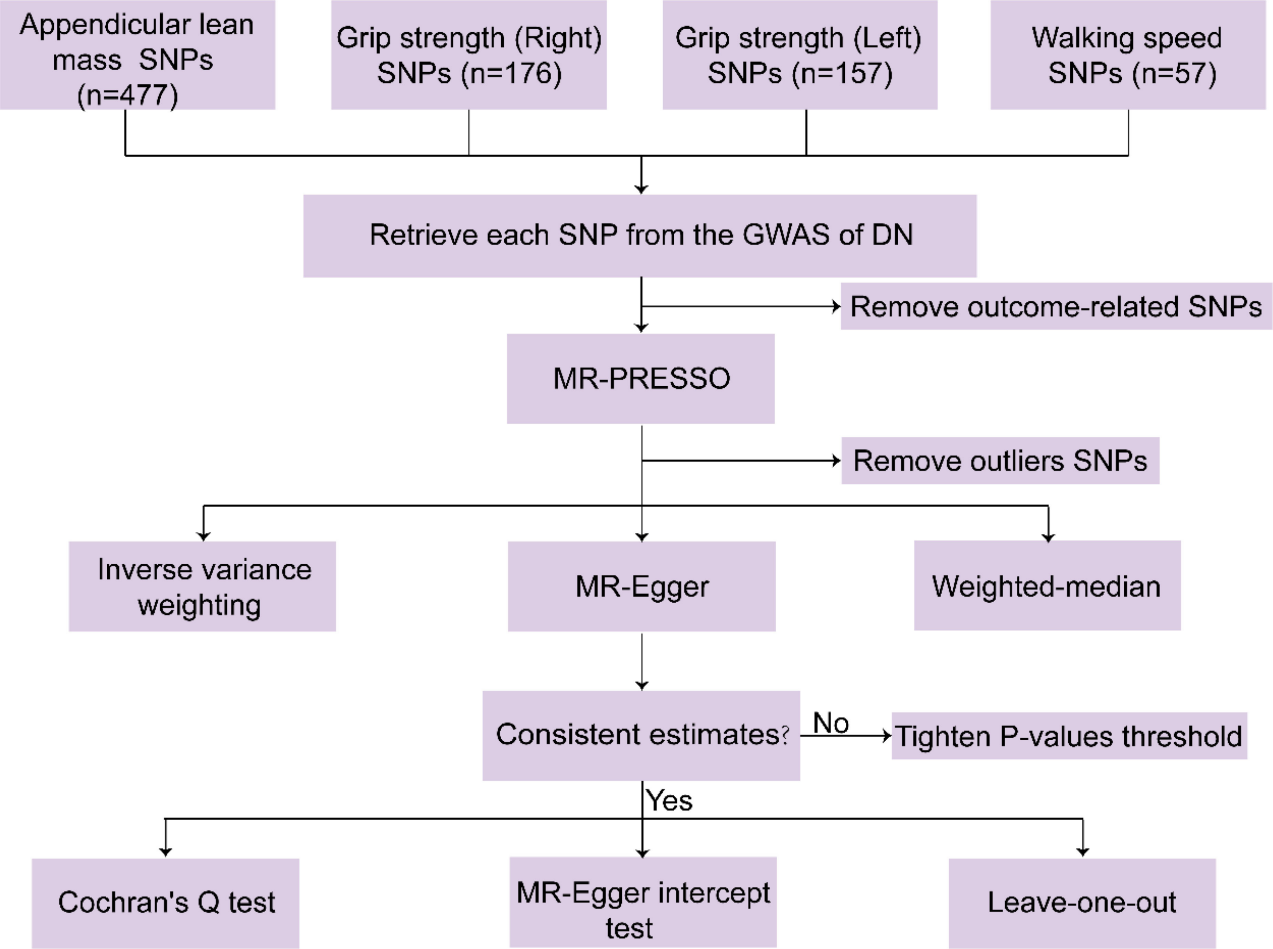
The framework diagram of the Forward MR study.

The summary statistics of SNPs related to appendicular lean mass, grip strength, walking speed were extracted from the GWAS database, which is publicly available. We used the following methods to select SNPs that fit the MR hypothesis: We set first P < 5 × 10^-8^ as the threshold for SNPs to reach genome-wide significance and [LD]r^2^ < 0.001 (clumping distance = 10,000 kb) as the cut-off value to determine whether that SNPs were in linkage equilibrium (21). Furthermore, weak IVs were excluded by calculating the F-value (the formula for calculating F was provided in the data analysis and data visualization section) (22). Second, after removing SNPs associated with DN at a threshold of 5 × 10^-8^ (23). The MR Pleiotropy RESidual Sum and Outlier (MR-PRESSO) method were used to eliminate potential outliers before each MR analysis (21). Finally, palindromic SNPs were eliminated by harmonizing exposure datasets with DN datasets (19, 24). After a thorough screening, the remaining SNPs were used for subsequent analyses. These SNPs are shown in detail in **Supplementary Materials 1–4** (**Supplementary Materials 1–4** showed SNPs for the effects of appendicular lean mass, grip strength (right), grip strength (left), and walking speed on DN, respectively). **Figure 2** displays the framework diagram of the study.

### Selection of instrumental genetic variables for diabetic nephropathy

**Figure 3:**
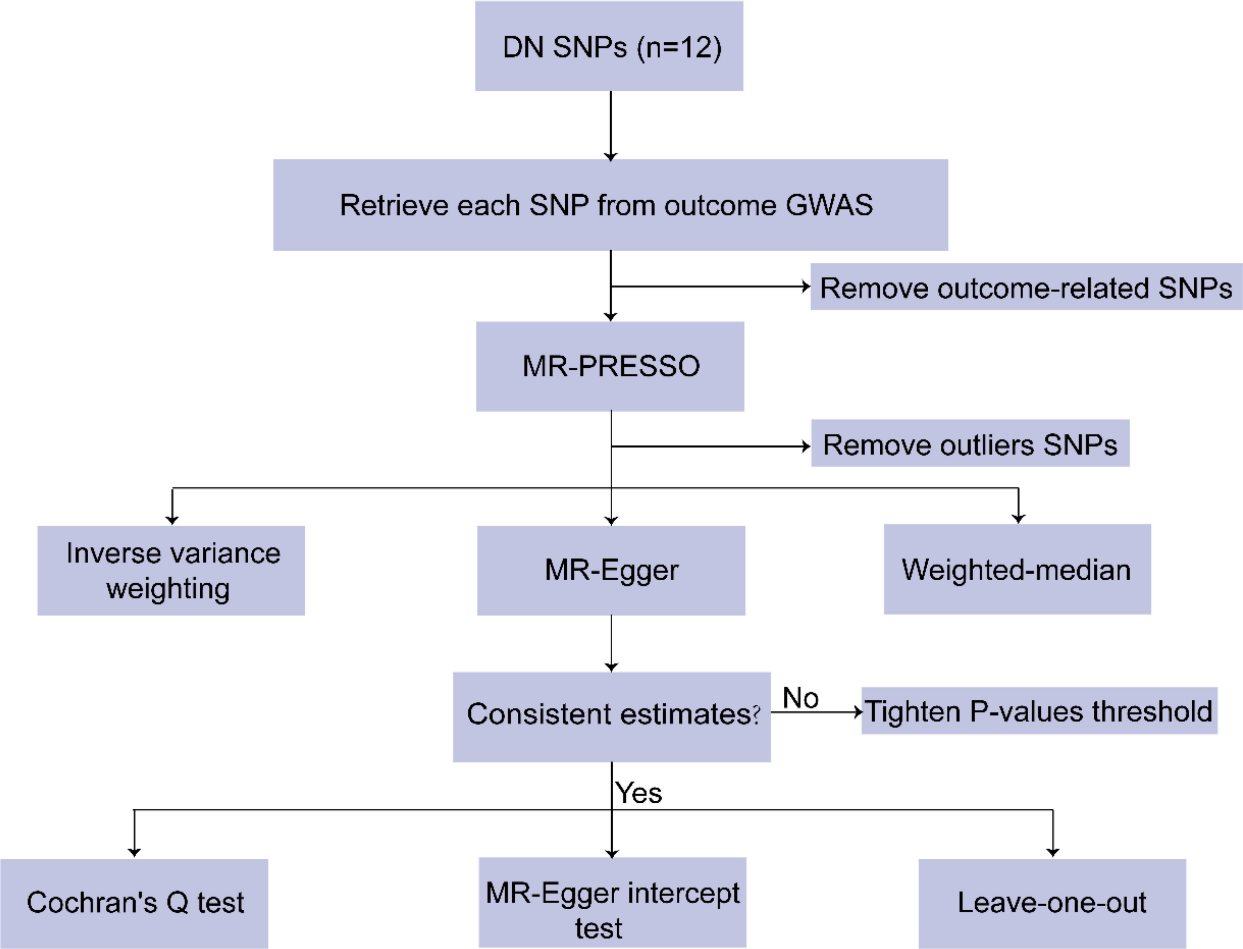
Flowchart of the Reverse MR study.

Summary statistics of SNPs associated with diabetic nephropathy were extracted from the GWAS, which included 3283 cases and 181,704 controls. The following steps were performed to screen out SNPs that met the MR hypothesis. First, we set P < 5 × 10^−6^ as the threshold for DN-associated SNPs, following the study of Cai et al. (25). Meanwhile, r^2^ < 0.001 (clumping distance = 10,000 kb) was set to exclude SNPs that were in linkage disequilibrium (21, 23). IVs with F < 10 were excluded (22). Second, after removing SNPs related to sarcopenia at a threshold of 5 × 10^-8^ (23). The MR-PRESSO was applied to remove the underlying outliers before each MR analysis (21). Finally, similar to Forward MR, the exposure and result datasets were reconciled (19, 24). After the above rigorous screening, the remaining SNPs were used in the subsequent analysis. These SNP data are visible in detail in Supplementary Material 5, 6, 7, and 8(**Supplementary Materials 5–8** showed the SNPs for the effects of DN on appendicular lean mass, grip strength (right), grip strength (left), and walking speed, respectively). The study frame diagram is presented in **Figure 3**.

### Statistical analysis and data visualization

The bias of the weak instrumental variables was evaluated by calculating the F-statistic. The formula for calculating F-statistic was consistent with Burgess et al. (22). A strong instrumental variable was F > 10 (22, 25). We used the MR-PRESSO method (1000 repeated settings) to find outliers (23, 26). Outliers were then eliminated so that the data could be re-evaluated. The randomized inverse variance weighting (IVW) was the primary statistical method for two-way MR analysis. We also performed MR Egger and weighted-median to ensure consistent results (23, 26). We performed a series of sensitivity analyses, including Cochran’s Q test, MR-Egger intercept test, and leave-one-out analysis, to measure the dependability of the results (23, 26). Heterogeneity was also determined using Cochran’s Q test (23, 26). Pleiotropy was evaluated using the MR-Egger intercept test and the Leave-one-out analysis (23, 26). The “TwoSampleMR” and “MR-PRESSO” packages of the R software (version 4.2.1) were used to implement all MR analyses as well as sensitivity analyses.

## Results

### The effect of sarcopenia on diabetic nephropathy

**Figure 4:**
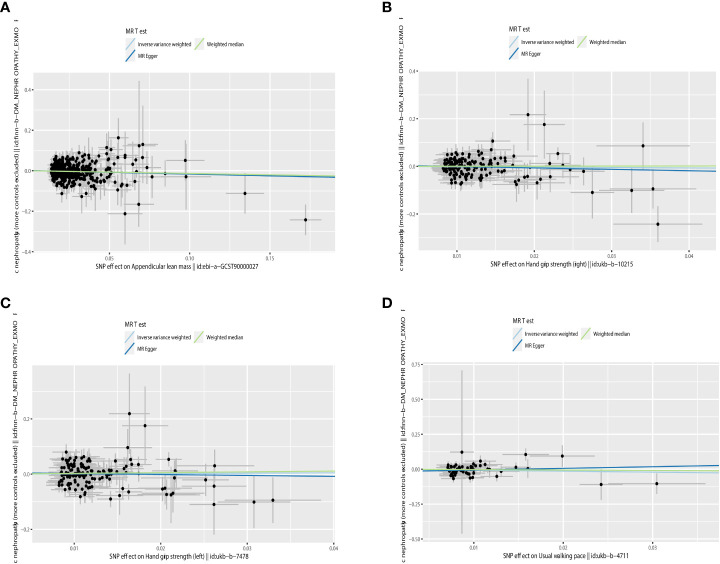
Scatter plot of the causality of sarcopenia on DN. **(A)** The causality of appendicular lean mass on DN. **(B)** The causality of grip strength (right) on DN. **(C)** The causality of grip strength (left) on DN. **(D)** The causality of walking speed on DN.

Increased appendicular lean mass was associated with a lower risk for developing DN among the three sarcopenic phenotypes examined, according to IVW analysis (OR = 0.863, 95% CI 0.767 - 0.971; P = 0.014). Other MR methods also production results similar to IVW, but were not statistically significant (26). Grip strength and walking speed had no causal impact on DN in the IVW analysis. Similar outcomes were obtained using the MR models, MR-Egger and Weighted-median (See **Table 2** and **Figure 4** for detailed results of MR analysis). Cochran’s Q test, MR-Egger intercept test and Leave-one-out analyses were conducted to measure the robustness and reliability of the above results. Cochran’s Q test P values were all >0.05, indicating that no heterogeneity was detected, except for P<0.05 for the grip strength (left), which had a P <0.05(**Table 2**). However, the random-effects IVW used as the primary in this investigation accepted heterogeneity (23). P > 0.05 was obtained for all MR-Egger intercept tests, suggesting the absence of horizontal pleiotropy (**Table 2**). Leave-one-out analyses showed that none of the SNPs significant impacted in the results, in other words, removing any SNPS would not have a significant effect on the results. Leave-one-out analyses were shown in **Supplement Material 9**.

**Table 2:** MR results from appendicular lean body mass, grip strength and walking speed on genetic prediction of DN.

Exposure	Outcome	nSNP	Method	OR	95%CI	P	Cochran’s Q-derived P value	MR-Egger intercept-derived P value
appendicular lean mass	DN	424	IVW	0.863	0.767-0.971	0.014	0.095	0.843
			MR-Egger	0.839	0.620-1.136	0.258		
			Weighted- median	0.859	0.716-1.031	0.103		
grip strength (right)	DN	164	IVW	0.847	0.552-1.300	0.447	0.082	0.600
			MR-Egger	0.561	0.114-2.768	0.478		
			Weighted- median	1.037	0.557-1.932	0.908		
grip strength (left)	DN	147	IVW	1.119	0.688-1.820	0.650	0.007	0.630
			MR-Egger	0.712	0.106-4.762	0.726		
			Weighted- median	1.316	0.697-2.482	0.397		
walking speed	DN	56	IVW	0.495	0.206-1.189	0.116	0.430	0.304
			MR-Egger	3.283	0.083-129.881	0.529		
			Weighted-median	0.745	0.210-2.641	0.648		

### The effect of diabetic nephropathy on sarcopenia

**Figure 5:**
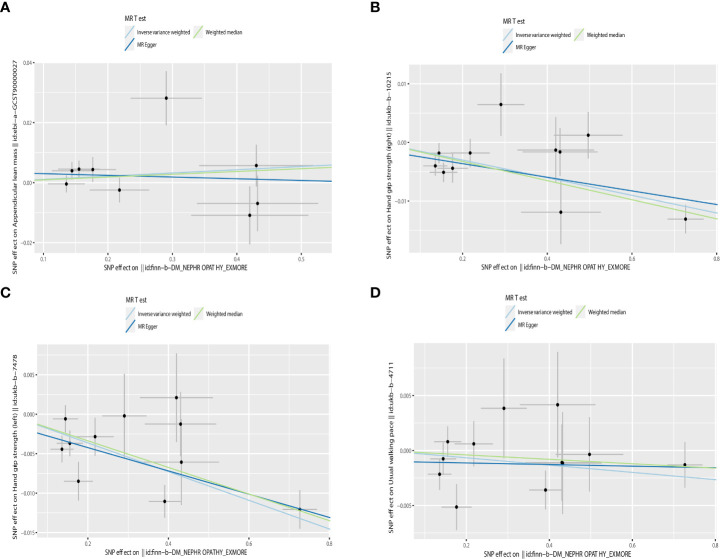
Scatter plot of the causality of DN on sarcopenia. **(A)** Scatter plot of the causality of DN on appendicular lean mass. **(B)** Scatter plot of the causality of DN on grip strength (right). **(C)** Scatter plot of the causality of DN on grip strength (left). **(D)** Scatter plot of the causality of DN on walking speed.

**Figure 6:**
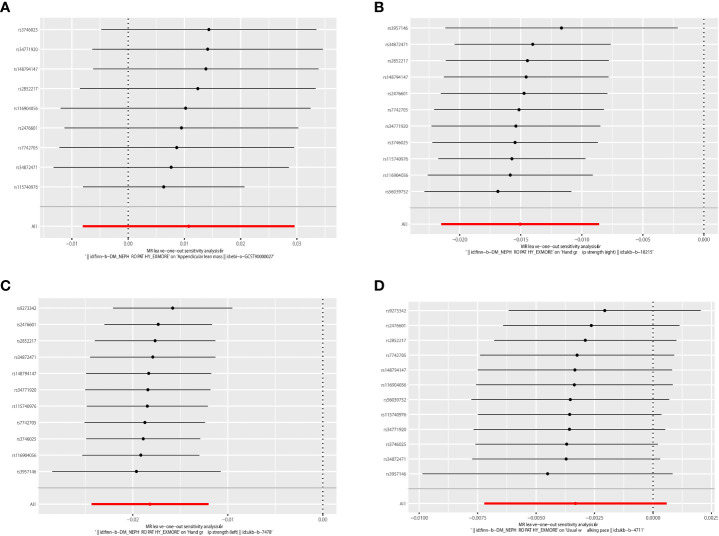
Leave-one-out of the effect of DN on sarcopenia. **(A)** Leave-one-out of the effect of DN on appendicular lean mass. **(B)** Leave-one-out of the effect of DN on grip strength (right). **(C)** Leave-one-out of the effect of DN on grip strength (left). **(D)** Leave-one-out of the effect of DN on walking speed.

IVW analysis showed that DN had a causal effect on grip strength (Right: β = 0.003, 95% CI: -0.021 to -0.009, P = 5.116e-06; Left: β = 0.003, 95% CI: -0.024 to -0.012, P = 7.035e-09). The weighted-median method produced similar results (Right: β = 0.003, 95% CI: -0.022 to -0.010, P = 1.258e-06; Left: β = 0.017, 95% CI: -0.023 to -0.011, P = 2.191e-07). MR-Egger showed a consistent but insignificant direction (Right: β=-0.012, 95% CI: -0.024 to 0.000, P=7.066e-02; Left: β = -0.015, 95% CI: -0.027 to -0.003, P=2.888e-02). In the IVW analysis, DN did not affect appendicular lean mass and walking speed. Other MR models, such as MR-Egger and Weighted-median, were consistent with the IVW findings (see **Table 3** and **Figure 5** for detailed results of MR analysis). Similarly, we performed Cochran’s Q test, MR-Egger intercept test, and Leave-one-out analysis to verify the reliability of reverse MR analysis results. All Cochran’s Q tests had P values were >0.05, indicating that no heterogeneity was found, except in the study of DN on appendicular lean body mass (**Table 3**). And because we used random effects IVW as the primary outcome, heterogeneity was acceptable (23). All MR-Egger intercept tests findings were P>0.05, suggesting no horizontal polymorphism was detected (**Table 3**). Eliminating one SNP did not change the directionality of the results, according to leave-one-out analyses. **Figure 6** displays leave-one-out analysis results.

**Table 3:** MR results from DN on genetic prediction of appendicular lean body mass, grip strength and walking speed.

Exposure	Outcome	nSNP	Method	Beta	95%CI	P	Cochran’s Q-derived P value	MR-Egger intercept-derived P value
DN	appendicular lean mass	9	IVW	0.011	-0.009, 0.031	0.263	0.049	0.497
			MR-Egger	-0.006	-0.055, 0.043	0.827		
			Weighted-median	0.009	-0.011, 0.029	0.228		
DN	grip strength (Right)	11	IVW	-0.015	-0.021, -0.009	5.116e-06	0.054	0.471
			MR-Egger	-0.012	-0.024, 0.000	7.066e-02		
			Weighted-median	-0.016	-0.022, -0.010	1.258e-06		
DN	grip strength (Left)	11	IVW	-0.018	-0.024, -0.012	7.035e-09	0.050	0.482
			MR-Egger	-0.015	-0.027, -0.003	2.888e-02		
			Weighted-median	-0.017	-0.023, -0.011	2.191e-07		
DN	walking speed	12	IVW	-0.003	-0.007, 0.001	0.096	0.360	0.406
			MR-Egger	-0.001	-0.009, 0.007	0.843		
			Weighted-median	-0.002	-0.008, 0.004	0.449		

## Discussion

We established the link between sarcopenia and DN in the current investigation. According to our Forward MR analysis, reducing in appendicular lean mass increases the risk of developing DN. Our Reverse MR analysis shows DN is linked to reduced grip strength. Therefore, there may be a causal relationship between sarcopenia and diabetic nephropathy on a single component basis. However, a stepwise diagnostic strategy must be used to diagnosis of sarcopenia rather than on one of these components (1). As a result, it is reasonable to say that there is no causal relationship between sarcopenia and DN.

Several observational studies produced varying outcomes. In a study involving 879 participants, Lin et al. discovered that patients with T2DM had increased visceral fate area and decreased lower extremity muscle mass as DN progressed (27). Additionally, DN was found to be a risk factor for the development of sarcopenia in diabetic patients, according to a recent meta-analysis (16). However, Ida S et al. discovered the exact reverse, demonstrating that sarcopenia was substantially linked to lower levels of urine albumin, urinary protein, and eGFR in patients with T2DM (17). Sarcopenia was also revealed to be a separate risk factor for DN in a recent study (15). Unfortunately, there wasn’t enough data from observational studies to prove that sarcopenia and DN weren’t related.

The following factors may be connected to the debate about sarcopenia and the risk of DN. Firstly, these investigations lacked randomization, prospective and blinding because they were observational research or meta-analyses built on observational studies. The shortcomings of the non-randomized comparative study itself may be to blame for the discrepancies in the results (19). Secondly, there may be variations in the sarcopenia diagnosis or measurement, which would explain the variations. For example, in the study by Huang et al., sarcopenia was defined as a skeletal muscle mass index two standard deviations or higher below the normal sex-specific mean for young adults (15). Asian Working Group on Sarcopenia criteria were used by Fung et al. to define sarcopenia as opposed to muscular mass, grip strength, and walking speed thresholds (28). Thirdly, sneaky confounding variables may be to blame for the association between sarcopenia and DN that observational studies have identified. Although numerous studies may have considered confounding factors like age, diabetes mellitus, and others, underlying confounding factors may still exist. Therefore, more research is required on the genetic link between DN and sarcopenia.

The main finding of our MR study is that there is no causal relationship between sarcopenia and DN. Our study provides several advantages. First, the MR study was a pioneer in determining how between sarcopenia and DN are causally related. The MR study is considered a natural RCT study with more reliable evidence than previous observational line studies (19). Second, the present study is limited to the European population, avoiding bias in population selection. Finally, the present study selected IVs based on appendicular lean mass, grip strength and walking speed, to assess the association with DN, which is more consistent with the current consensus on the development of sarcopenia diagnosis in Europe and Asia (2, 3).

Our MR analysis has some limitations, which will need to be fixed. First, a larger GWAS database of DN may be required to confirm causation due to the limited sample size of DN and the relatively poor statistical power of MR analysis. Second, the study suggests that sarcopenia may be influenced by age and sex (29). In contrast, GWAS data lack age and gender stratification to analyze the cause and effect of sarcopenia and DN in various subgroups in more detail. Third, no DN subtype was present in the GWAS data to measure the relationship between sarcopenia and different DN subtypes.

In conclusion, the current bidirectional MR study demonstrate the causal relationship between sarcopenia and DN is complex and variable, which is related to the diagnostic criteria of sarcopenia. Additional studies based on larger GWAS or age-stratified MR studies are required to validate these findings.

## Data availability statement

The original contributions presented in the study are included in the article/**Supplementary Material**. Further inquiries can be directed to the corresponding authors.

## Ethics statement

Written informed consent was obtained from the individual(s) for the publication of any potentially identifiable images or data included in this article.

## Author contributions

LR, XG, and GW contributed to the study conception, design, and manuscript drafting. YW, FJ, and MS contributed to the acquisition and analysis of data. All authors contributed to the article and approved the submitted version.
