# Supplementary figures and images for: Causality between sarcopenia and diabetic nephropathy: a bidirectional Mendelian randomization study
Source: Front Endocrinol (Lausanne). 2023 May 22;14:1188972. doi: 10.3389/fendo.2023.1188972 (PMC10239922; doi:10.3389/fendo.2023.1188972)

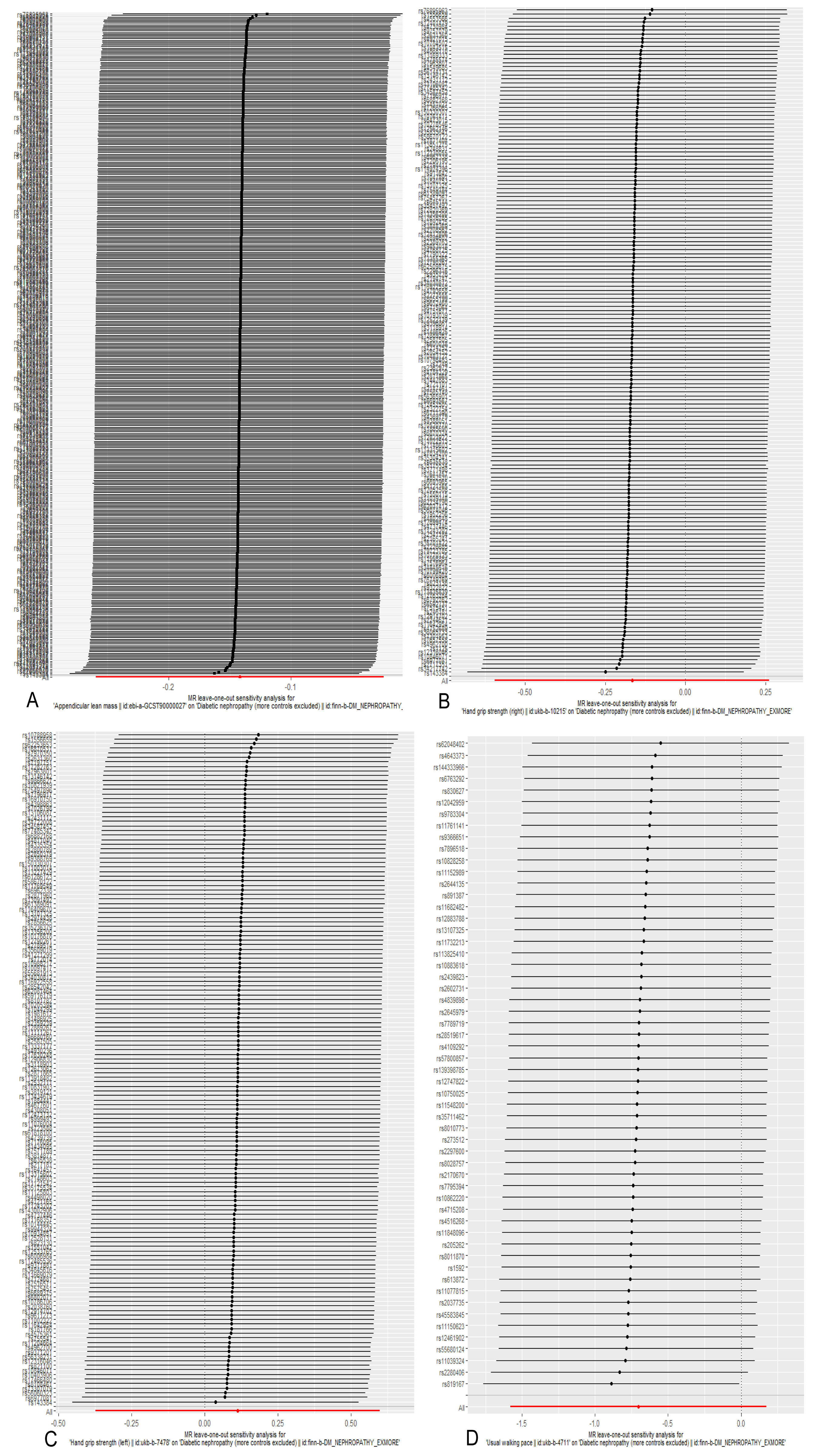

Supplement: Supplementary file 9 [file Image_1.tif]
